# Feasibility of the Impression Reference (IR) Technique for Open-Flap Immediate Loading Full-Arch Rehabilitation of the Atrophic Maxilla: A Case Series

**DOI:** 10.3390/jcm15155903

**Published:** 2026-07-28

**Authors:** Gerardo Pellegrino, Carlo Barausse, Subhi Tayeb, Martina Sansavini, Alessandro Antonelli, Cristiana Breccia, Pietro Di Bene, Pietro Felice

**Affiliations:** 1 Oral Surgery Unit, Department of Biomedical and Neuromotor Sciences (DIBINEM), University of Bologna, 40125 Bologna, Italy; carlo.barausse@unibo.it (C.B.); subhi.tayeb@unibo.it (S.T.); martina.sansavini@studio.unibo.it (M.S.); pietro.felice@unibo.it (P.F.); 2 Department of Health Sciences, Magna Graecia University of Catanzaro, 88100 Catanzaro, Italy; alessandro.antonelli@unicz.it; 3 Prosthodontic Unit, Department of Biomedical and Neuromotor Sciences (DIBINEM), University of Bologna, 40125 Bologna, Italy; breccia.cristiana@gmail.com; 4 Nexxta S.p.A, 40127 Bologna, Italy; p.dibene@nexxtaspa.com

**Keywords:** impression reference, immediate loading, full-arch rehabilitation, atrophic maxilla, open-flap surgery, digital workflow

## Abstract

**Background:** Open-flap immediate loading full-arch rehabilitation of the atrophic edentulous maxilla remains clinically demanding because flap elevation modifies the soft-tissue anatomy and stable anatomical landmarks for digital registration are limited. The Impression Reference (IR) technique was developed as a workflow-oriented strategy to provide a recognizable spatial reference during dataset matching and prosthetic transfer. This case series evaluated the feasibility of completing an IR-assisted digital workflow in patients undergoing open-flap immediate full-arch rehabilitation of the atrophic maxilla. **Methods:** This feasibility case series included 26 completely edentulous patients with severe maxillary atrophy treated with immediate full-arch implant-supported rehabilitation using an Impression Reference-assisted digital workflow. No control group was included and no quantitative accuracy analysis was performed. Workflow-related and prosthetic-transfer outcomes were evaluated descriptively, including completion of dataset matching, prosthetic transfer, IR coupling within the predefined 0.2 mm tolerance, delivery of the immediate prosthesis, clinically acceptable passive fit, prosthetic congruence with the pre-surgical digital plan, and surgical complications. **Results:** The IR-assisted workflow, dataset matching, prosthetic transfer, and immediate prosthetic delivery were completed in all 26 patients. All patient-specific digital projects complied with the nominal 0.2 mm CAD coupling tolerance, and the associated components could be completely seated and remained clinically stable during use. Clinically acceptable prosthesis fit and prosthetic congruence with the pre-surgical project were recorded in all immediate restorations according to the predefined clinical criteria. No intraoperative surgical complications were documented in the available records. **Conclusions:** Within the limitations of this descriptive case series, the IR-assisted workflow was feasible in all the included patients undergoing open-flap immediate loading full-arch rehabilitation of the atrophic maxilla. Successful workflow completion should not be interpreted as quantitative evidence.

## 1. Introduction

Immediate loading implant-supported full-arch rehabilitation is increasingly used in the treatment of edentulous patients, as it may shorten treatment time and provide early functional and psychosocial benefits [1,2]. However, its clinical application remains demanding, particularly in patients with severe maxillary atrophy, complex surgical requirements or open-flap approaches [3,4]. In these situations, the surgical and prosthetic phases are strictly interconnected, and the accurate transfer of information throughout the workflow becomes a key prerequisite for achieving a predictable restorative outcome [5,6,7]. The progressive integration of digital technologies into implant prosthodontics has significantly improved treatment planning and prosthetic manufacturing. Intraoral scanning, cone-beam computed tomography (CBCT), and CAD/CAM systems may facilitate the creation of a virtual patient and support a more efficient restorative sequence [8,9,10]. Nevertheless, the advantages of a digital workflow depend on the possibility of reliably aligning radiographic, soft-tissue, prosthetic, and implant-related information [11,12]. This step may be relatively standardized in flapless guided procedures or when stable anatomical references are available, but it becomes more challenging in extensive open-flap surgery and in completely edentulous maxillae. Under these conditions, the absence of natural dental landmarks and the modification of soft-tissue anatomy during surgery may reduce the predictability of dataset matching and make the digital transfer more technique-sensitive [13,14,15]. In such cases, rehabilitation may require different implant strategies according to the residual anatomy and the planned prosthetic design. Zygomatic and pterygoid implants may represent valuable options when conventional implant placement is limited or not feasible without extensive grafting procedures [16,17,18,19,20,21,22,23,24]. In other clinical scenarios, standard, short, extra-short, or narrow implants may be selected to support immediate fixed rehabilitation [25,26,27]. More broadly, atrophic edentulous maxillae may require immediate full-arch rehabilitation protocols in which maintaining spatial coherence between planning, surgery, and prosthetic delivery is essential [28,29,30]. In this context, the main limitation of a purely digital workflow is often not the acquisition of isolated datasets itself, but rather the possibility of maintaining a stable and recognizable reference throughout the different clinical and laboratory phases. To address these difficulties, a digital workflow based on the use of an impression reference has previously been proposed [31]. The technique was initially introduced to simplify the prosthetic sequence of immediate loading rehabilitation by facilitating the alignment of stereolithographic files and radiographic datasets and by supporting the registration of preoperative and postoperative scans. The rationale of the method is based on the use of a prototyping reference device with predefined points, angles, and bushings, designed to provide a stable and identifiable reference during the digital transfer process. From a clinical point of view, this approach may be particularly useful in completely edentulous maxillae, where the operator cannot rely on natural teeth as constant landmarks for orientation. More recently, the same concept has been extended to open-flap digital workflows for immediate loading rehabilitation [32]. The present investigation does not introduce the Impression Reference concept for the first time. Rather, it extends the available evidence beyond individual technical reports by describing the clinical application of the third-generation configuration across a series of atrophic maxillae treated with different implant-supported rehabilitation strategies. In this configuration, the core Impression Reference has a standardized geometry, while patient-specific adaptation is achieved through customized positioning, inspection, and connecting components derived from the validated prosthetic project. The primary aim of this case series was to evaluate the clinical feasibility of completing an Impression Reference-assisted digital workflow for open-flap immediate loading full-arch rehabilitation of the atrophic edentulous maxilla. Feasibility was assessed in terms of successful completion of the planned workflow, including intraoperative digital acquisition, dataset matching, prosthetic transfer, and delivery of an immediate screw-retained full-arch restoration. The study was not designed to provide quantitative measurements of dataset-matching accuracy, implant positional deviation, prosthetic misfit, or long-term clinical effectiveness.

## 2. Materials and Methods

### 2.1. Study Design and Patient Selection

This study was designed as a clinical workflow feasibility case series. It included all completely edentulous patients presenting with maxillary atrophy who required an open-flap approach for immediate implant-supported full-arch rehabilitation and were treated using the Impression Reference-assisted digital workflow between 2021 and 2025 at the Oral Surgery and Implantology Unit and the Prosthodontic Unit, Department of Biomedical and Neuromotor Sciences, University of Bologna. The need for an open-flap surgical approach represented the principal clinical criterion for inclusion. Patients presented with different degrees and patterns of maxillary atrophy and were treated using different implant-supported strategies according to the residual bone anatomy and prosthetic requirements, including zygomatic, pterygoid, standard, short, extra-short, and narrow implants. Severe maxillary atrophy was primarily observed in patients requiring zygomatic implant-supported rehabilitation, who represented the majority of the cohort. However, a uniform anatomical severity classification had not been prospectively recorded for all patients. Therefore, the broader term “atrophic maxilla” was adopted to describe the overall study population. Patients were eligible when they were completely edentulous in the maxilla, required open-flap surgery, underwent immediate full-arch rehabilitation using the Impression Reference-assisted workflow, and had the clinical, radiographic, and digital records required to document the prespecified workflow steps. During the study period, 26 patients fulfilled these eligibility criteria and were included in the case series. No eligible patients were excluded. Patients were not selected on the basis of rehabilitation protocol, successful workflow completion, prosthetic outcome, or occurrence of complications. All patients received detailed information regarding the surgical and prosthetic procedures and provided written informed consent before treatment.

### 2.2. Digital Workflow

**Figure 1:**
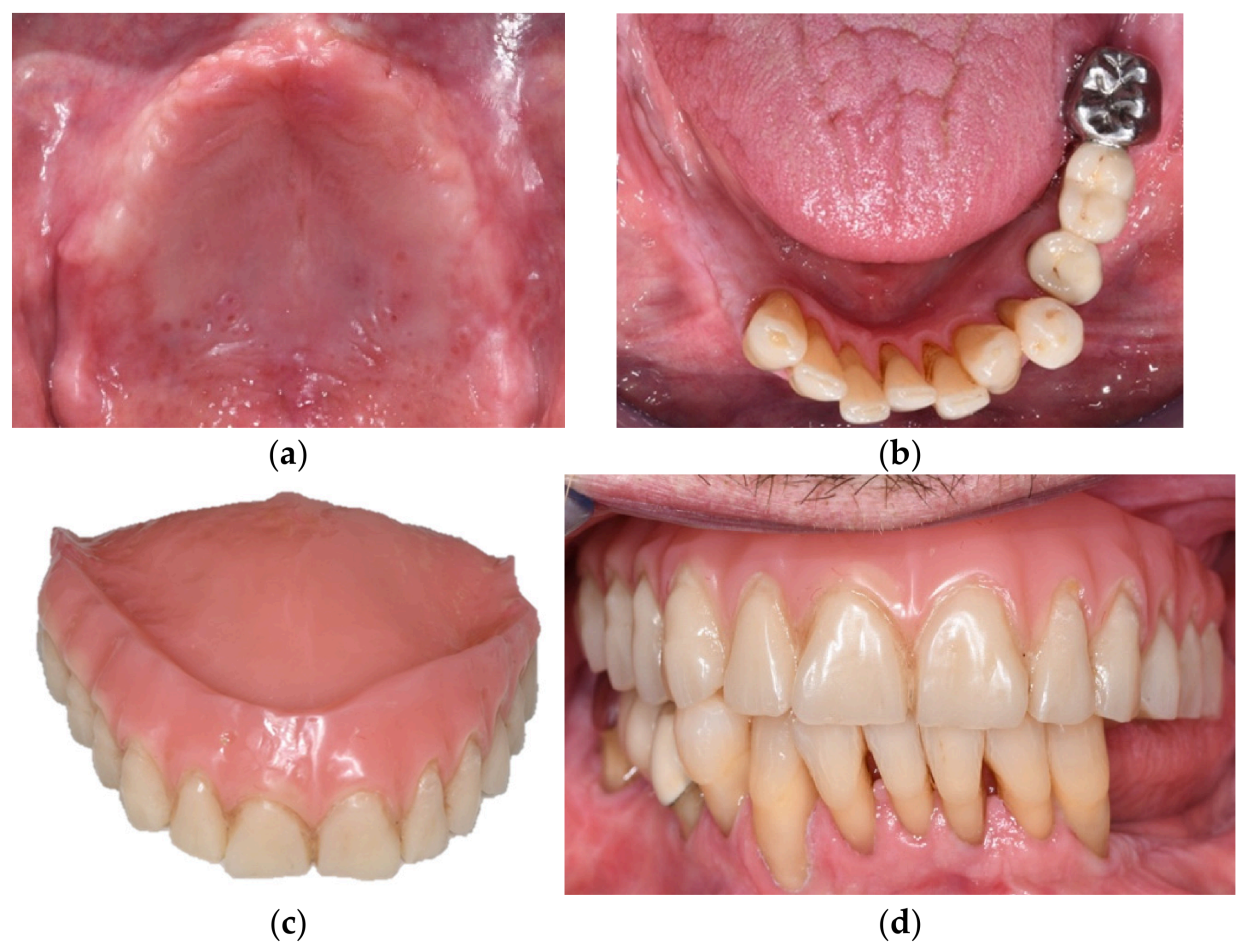
Preoperative clinical and prosthetic assessment. (**a**) Clinical evaluation of the completely edentulous maxillary arch and supporting tissues. (**b**) Evaluation of the opposing arch and available occlusal references. (**c**) Extraoral assessment of the patient’s pre-existing removable maxillary prosthesis. (**d**) Intraoral verification of the prosthesis, including adaptation, stability, vertical dimension, occlusal relationships, tooth position, midline, smile line, and lip support.

A digital workflow was adopted to generate an integrated virtual patient for prosthetically driven full-arch treatment planning. The process started from the patient’s pre-existing removable prosthesis, which was used as the basis for the fabrication of a diagnostic prosthetic prototype (Figure 1a–d). This prototype was designed to reproduce the planned full-arch rehabilitation in terms of tooth position, vertical dimension, occlusal scheme, soft-tissue support, midline, smile line, and esthetic–functional relationships. 

**Figure 2:**
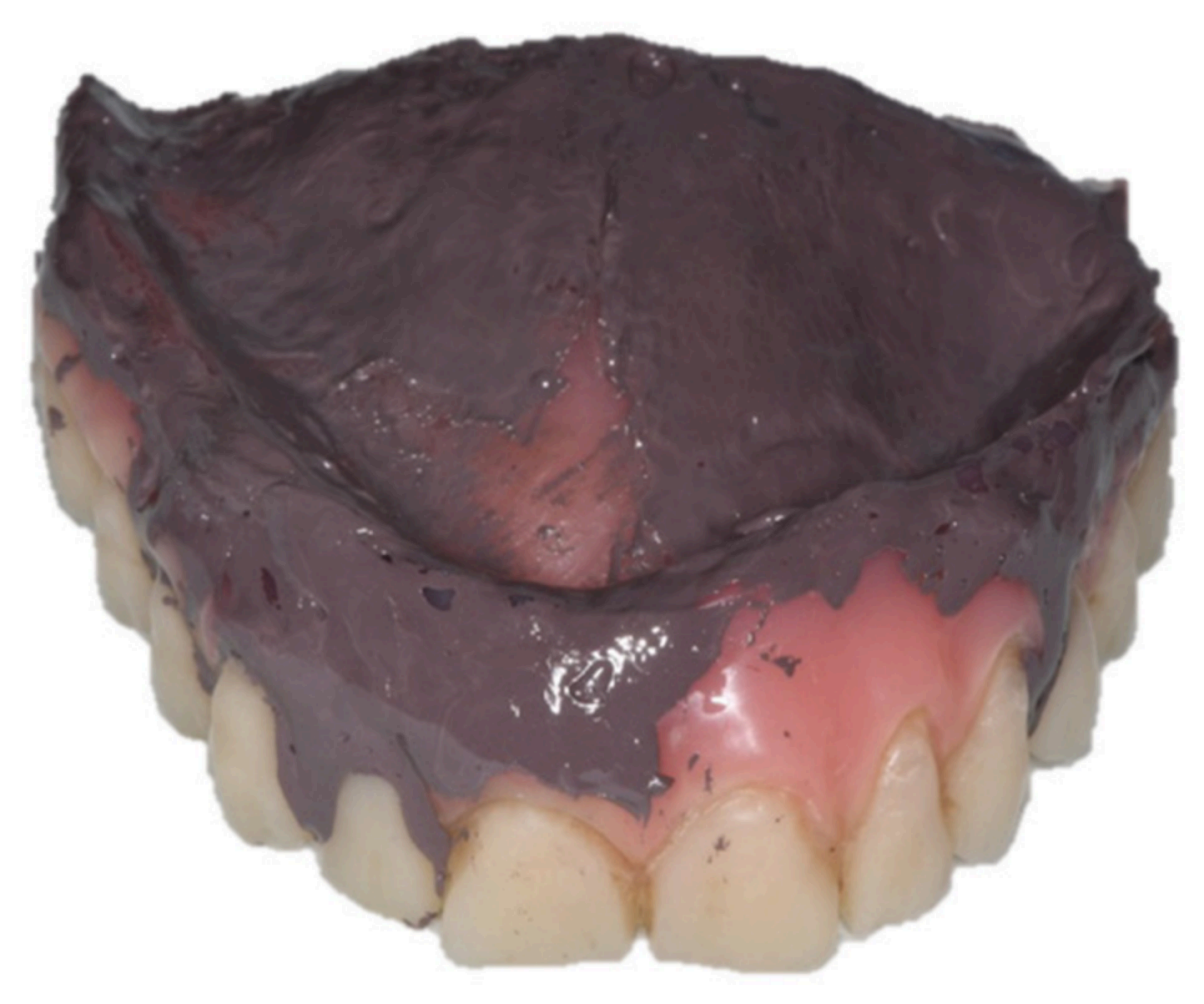
Relining of the removable prosthesis to improve adaptation and stability on the edentulous supporting tissues before digital acquisition.

Thus, implant planning was not performed as the initial step, but rather derived from a prosthetic project that had been preliminarily defined and clinically validated. When the adaptation of the prototype to the edentulous supporting tissues was not considered adequate, a relining procedure was performed before data acquisition (Figure 2).

**Figure 3:**
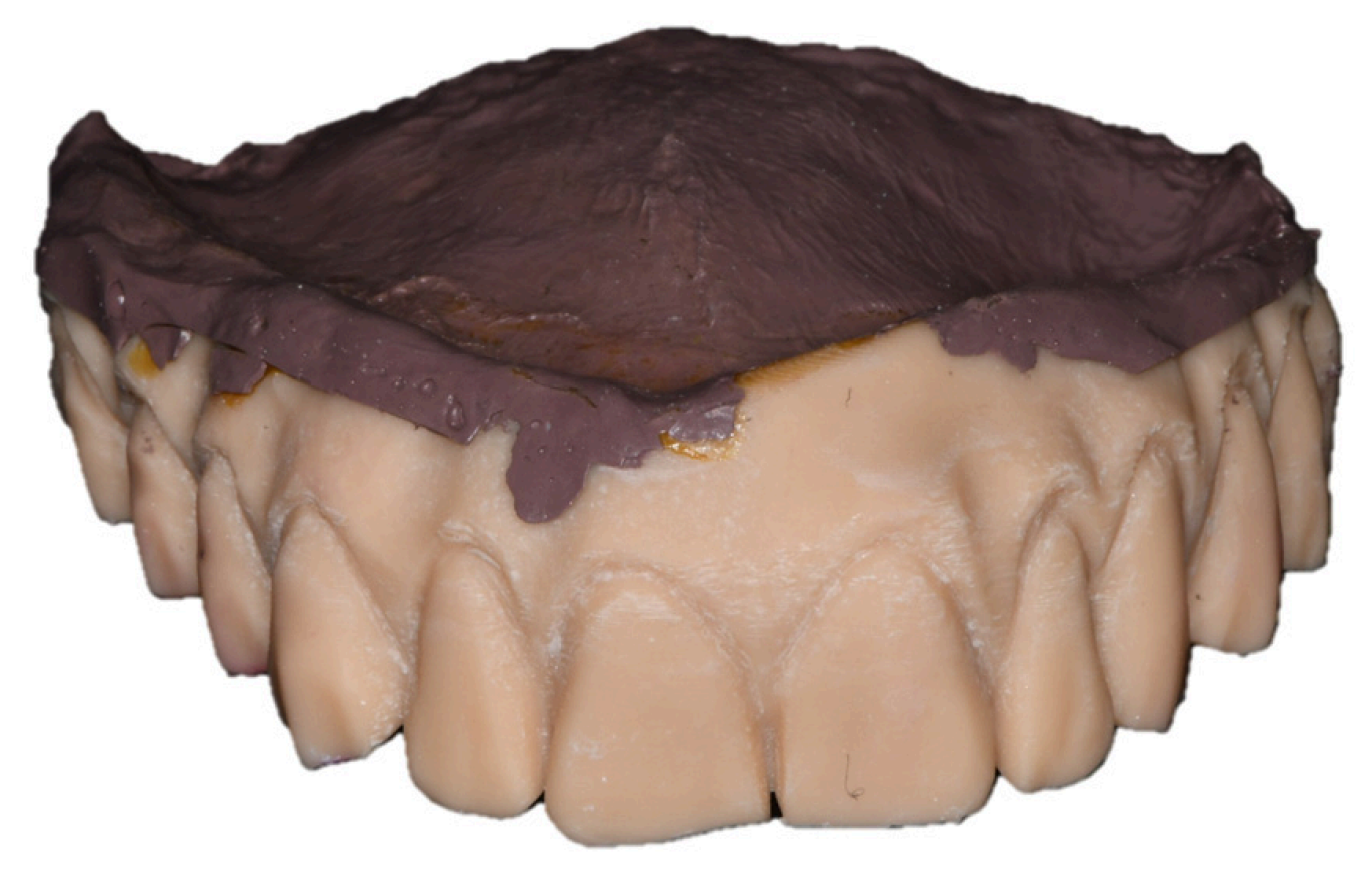
Duplication of the clinically validated prosthetic prototype and fabrication of the radiographic template for the double-scan CBCT protocol.

Once clinically satisfactory adaptation had been achieved, the prosthetic setup was re-evaluated intraorally, with particular attention to stability, vertical dimension, occlusal scheme, tooth position, midline, smile line, and lip support. Any required corrections were completed at this stage, so that the subsequent steps could be based on a validated prosthetic project. The approved prototype was then digitized extraorally using an intraoral scanner. The opposing arch and the maxillomandibular relationship were also recorded to reproduce the occlusal arrangement within the digital workflow. Subsequently, the prototype was duplicated and converted into a radiographic template (Figure 3). 

**Figure 4:**
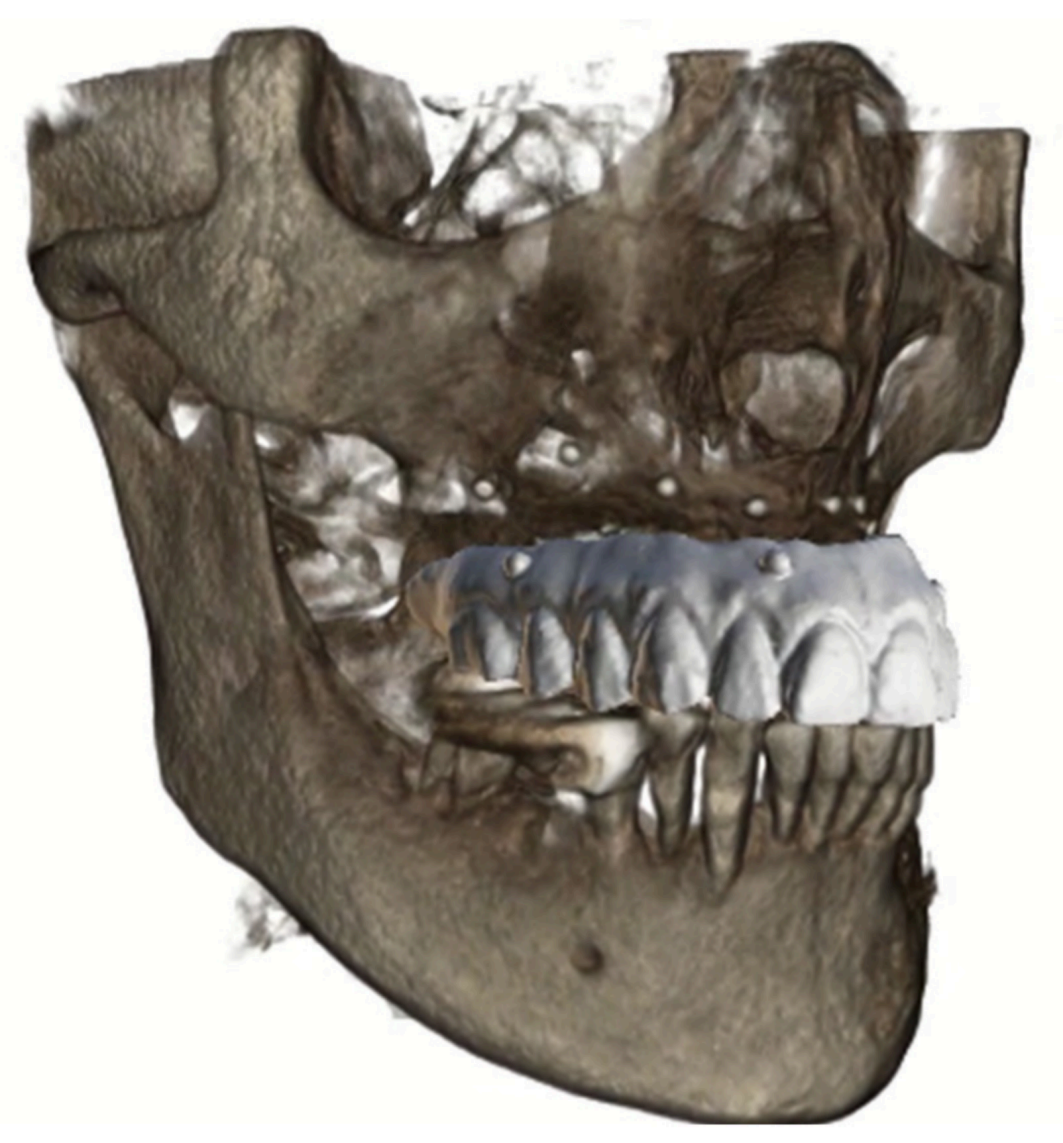
Registration of the CBCT, radiographic-template, prosthetic, soft-tissue, and occlusal datasets to create the integrated virtual patient and perform prosthetically driven implant planning.

Quartz radiopaque markers were applied to the outer surface of the template to facilitate the subsequent registration between radiographic and prosthetic datasets. A double-scan CBCT protocol was used: a first scan was acquired with the radiographic template seated in the patient’s mouth and maintained in occlusion, while a second scan was performed on the template alone. The radiographic dataset of the template could also be aligned with the STL file obtained from the extraoral 360-degree scan of the prosthesis or prosthetic prototype, in order to improve the definition of the prosthetic contours and increase the accuracy of digital registration (Figure 4). All acquired datasets were imported into the planning software and registered within the same digital project. 

**Figure 5:**
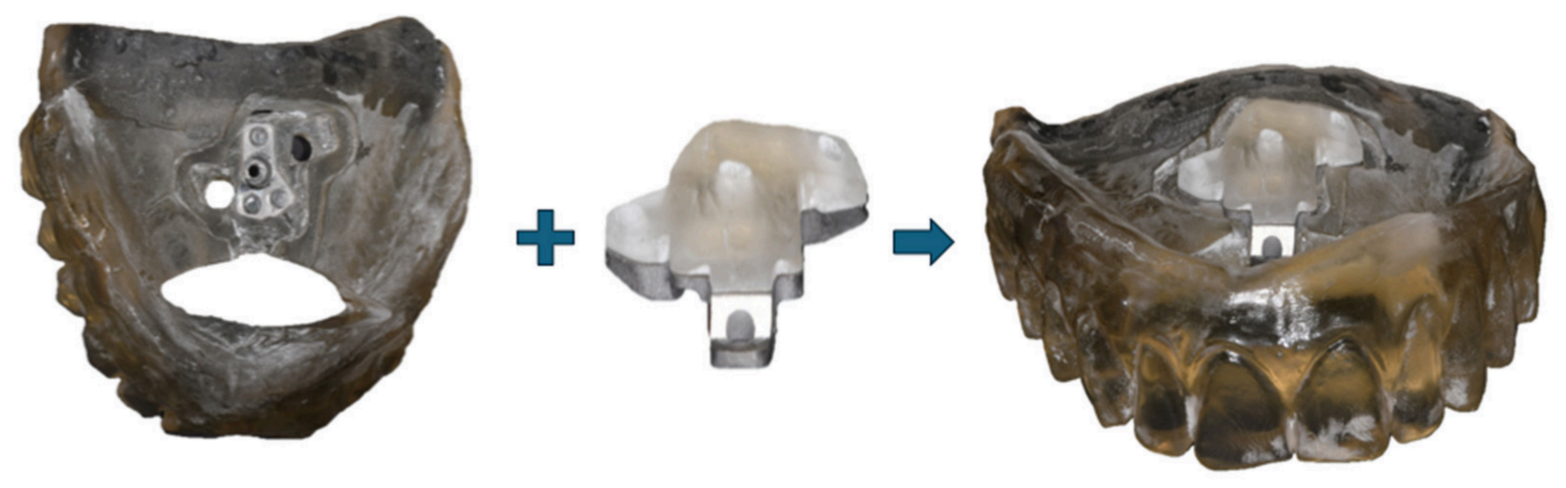
CAD design of the Impression Reference and positioning guide based on the validated prosthetic project and planned intraoral position.

The combination of radiographic, prosthetic, occlusal, and soft-tissue information allowed for the creation of an integrated virtual representation of the patient, including skeletal anatomy, soft-tissue morphology, high-definition prosthetic contours, and occlusal relationships. This virtual patient was then used for full-arch implant planning, guided by the planned prosthetic emergence and the previously validated restorative design. The Impression Reference was subsequently designed on the basis of this integrated digital project, before proceeding with the surgical and prosthetic phases of the workflow (Figure 5).

### 2.3. Impression Reference Technique: Design, Generations, and Fabrication

The impression reference was designed as a spatial transfer device to support the digital transfer of information in open-flap, immediately loaded full-arch rehabilitations of the edentulous maxilla. Its purpose was to provide a stable and recognizable geometry during the different registration steps of the workflow, rather than to act as an independent surgical guide. The device was developed after creation of the virtual patient and preliminary prosthetically driven implant planning, and its position was defined according to the planned prosthetic volume, the expected implant emergence areas, and the anatomy of the atrophic maxilla. 

The impression reference concept has progressively evolved through three generations. 

**Figure 6:**
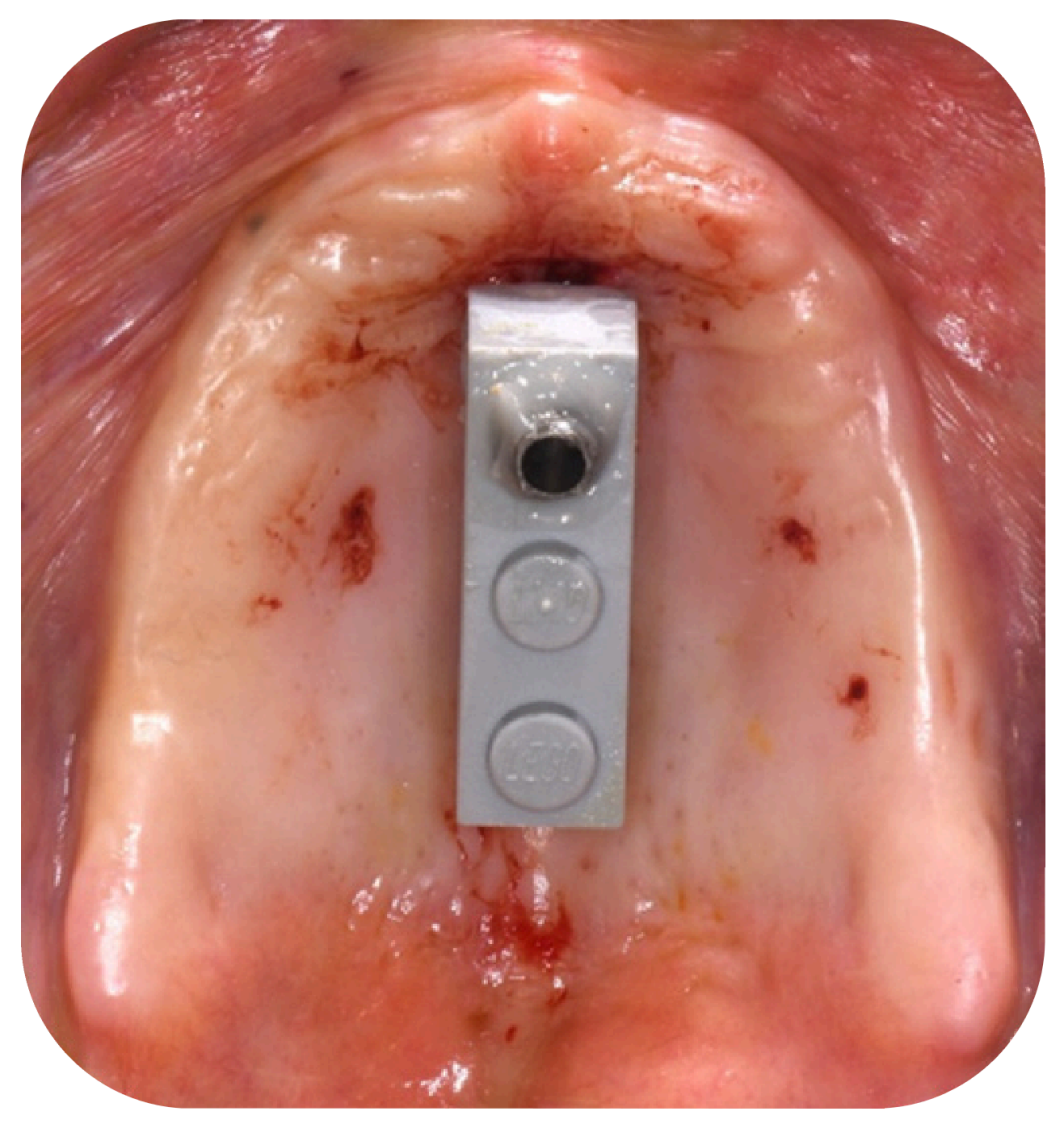
First-generation IR.

The first-generation impression reference was introduced as a spatial reference device for re-matching pre-surgical prosthetic designs with postoperative digital acquisitions. In this initial configuration, the device was mainly used to reconnect the preoperative prosthetic project, including smile anatomy and planned restorative contours, with the post-surgical intraoral dataset (Figure 6).

**Figure 7:**
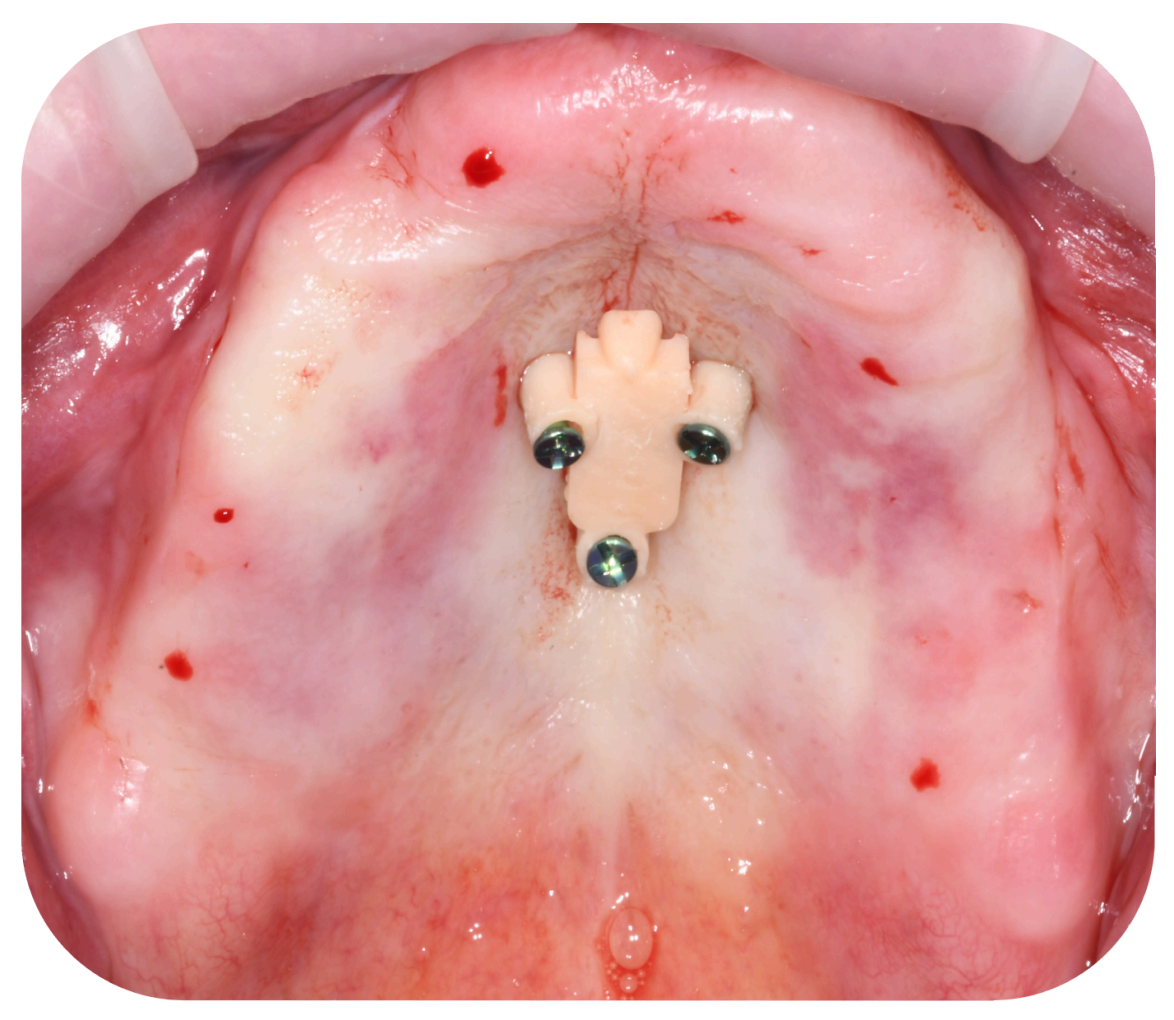
Second-generation IR.

The second-generation impression reference introduced a more guided transfer concept. In this configuration, the device was assigned a predefined intraoral position during the digital planning phase, and this planned position was transferred clinically using a dedicated positioning guide. Once positioned, the impression reference could be connected to an inspection guide, allowing the clinician to verify the prosthetic–implant relationship, including the orientation of the multi-unit abutments and the position of implant access channels or guide holes (Figure 7). 

**Figure 8:**
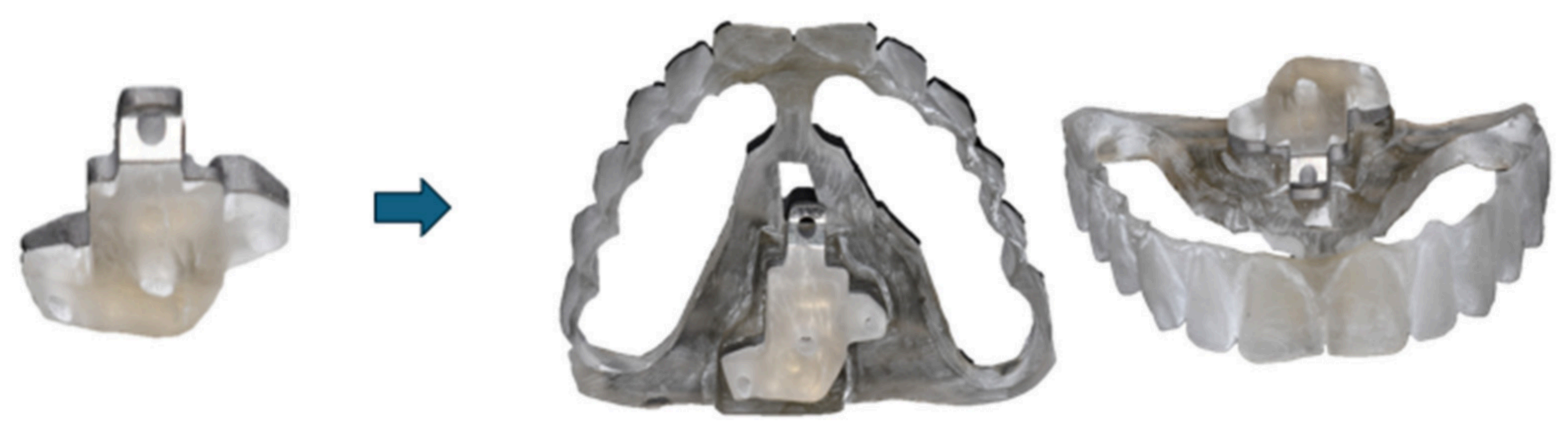
Third-generation IR.

The third-generation impression reference, used in the present case series, was developed to simplify and standardize the workflow. Unlike previous patient-specific configurations, this device has a standardized design that does not change from patient to patient. It can be used either as a free spatial reference, similarly to the first-generation approach, or as part of a guided workflow comparable to the second-generation configuration. When used within the guided workflow, the standardized impression reference can be associated with a positioning guide and an inspection guide. In this configuration, a case-specific customized adapter is designed to compensate for the spatial discrepancy between the standardized reference device and the individual prosthetic or soft-tissue morphology (Figure 8). This allows the same standardized reference device to be adapted to the patient-specific prosthetic anatomy without modifying the impression reference itself. All reference components and related guides were derived from the digital segmentation of the provisional removable prosthesis. This prosthesis was clinically validated before surgery to define the aesthetic parameters, smile line, lip support, prosthetic volume, occlusal relationships, and restorative architecture. After 360-degree scanning, the prosthetic volume was digitally separated and used to generate the Impression Reference-related components, including the positioning guide, the inspection guide, and, when required, the customized adapter. 

The impression reference was designed using DentalCAD 2.4 software (exocad, Darmstadt, Germany) and manufactured with a digital light processing three-dimensional printer (Model 5100 printer/software, 1920 × 1080 resolution; NextDent 3D Systems, Soesterberg, The Netherlands). The digital information used for this phase derived from the validated prosthetic prototype, acquired with an intraoral scanner (TRIOS; 3Shape, Copenhagen, Denmark), converted into a radiographic template using Model 2.0 resin (NextDent 3D Systems, Soesterberg, The Netherlands), and integrated with radiopaque quartz markers for the double-scan protocol. Radiographic data were obtained with a CBCT unit (NewTom, Cefla, Imola, Italy), and the matching procedure was performed using implant-planning software (BlueSkyBio 4.0, New York, NY, USA). During the clinical workflow, the impression reference was planned for fixation at the palatal midline. When guided positioning was required, the positioning guide reproduced the prosthetic project and included a dedicated housing for controlled seating of the reference device according to the digital plan. The connection between the components was obtained through a snap-on attachment system (OT; Rhein 83, Bologna, Italy), allowing stable and reproducible transfer of the impression reference from the digital project to the operative field. After seating, the device was stabilized with 8 or 11 mm bone screws (Global D, Brignais, France). The inspection guide, also referred to as an open guide, reproduced the external profile of the planned definitive restoration and could be connected to the impression reference to verify the relationship between the planned prosthetic contours, implant emergence areas, multi-unit abutment orientation, and implant access channels or guide holes. A nominal coupling tolerance of 0.2 mm was incorporated during the CAD design phase between the standardized Impression Reference and the associated positioning, inspection, or customized connecting components. Before manufacturing, each patient-specific digital project was verified to confirm compliance with this predefined design requirement. The tolerance was intended to facilitate complete seating and reproducible assembly while limiting excessive movement between the coupled components. This value represented a CAD design parameter and was not a clinical or metrological measurement of the physical discrepancy between the manufactured components, dataset-matching accuracy, prosthetic fit, or implant positional accuracy.

### 2.4. Surgical Procedure

**Figure 9:**
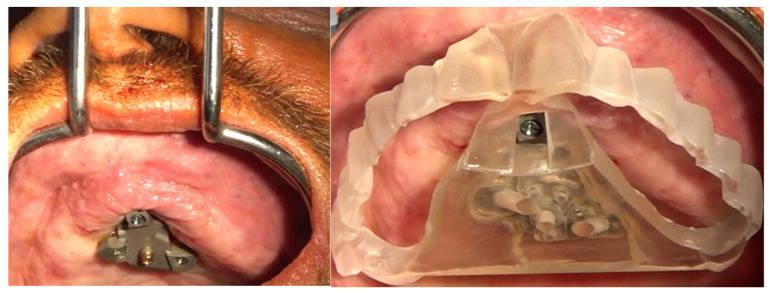
Fixation of the IR.

All surgical procedures included in the present case series were performed in the edentulous maxilla using an open-flap approach. Under local anesthesia, the impression reference was seated with the dedicated positioning stent and stabilized at the palatal midline with 8 or 11 mm bone fixation screws (Global D, Brignais, France). After fixation, the impression reference provided a stable reference for the intraoperative digital acquisition and for the subsequent alignment procedures required for prosthetic transfer (Figure 9). Before flap elevation, an intraoral scan of the edentulous maxilla was acquired with the impression reference in situ, in order to record its spatial relationship with the mucosal anatomy and the prosthetic project. A full-thickness maxillary flap was then elevated to expose the surgical field and allow implant placement under direct vision. The subsequent surgical sequence was adapted according to the planned rehabilitation protocol. All surgical procedures were performed by the same experienced surgeon. The intraoral scanning, dataset-matching, and prosthetic workflow procedures were performed by the same experienced prosthodontist. The surgical and prosthetic-digital roles were therefore assigned to two different clinicians, and this operator allocation remained consistent across all included patients.

#### 2.4.1. Zygomatic Implant Rehabilitation

In patients treated with zygomatic implant-supported rehabilitation, the impression reference was fixed at the palatal midline and the initial intraoral scan was acquired before flap elevation. A full-thickness maxillary flap was raised through a crestal incision with distal releasing incisions, while preserving the maxillary tuberosity. Implant site preparation was performed according to the planned zygomatic protocol. The customized open guide was connected to the impression reference and used to verify the relationship between the planned prosthetic contours, implant entry points, and expected emergence areas. Four zygomatic implants were then inserted along the planned trajectories. When required by the prosthetic design, additional conventional implants were placed in the anterior or premolar maxillary regions to support the immediate full-arch restoration. After implant placement, conical abutments were connected and healing caps were positioned to facilitate suturing and preserve the prosthetic references for the following digital acquisition phase.

#### 2.4.2. Pterygoid Implant Rehabilitation

In patients requiring posterior maxillary anchorage, the impression reference was fixed at the palatal midline before flap elevation. After acquisition of the initial intraoral scan with the device in place, a full-thickness flap was elevated to expose the posterior maxillary region and allow direct surgical access. The open guide was connected to the impression reference to maintain the spatial relationship with the planned prosthetic contours and to assist in verifying the intended implant emergence. Pterygoid implant site preparation and implant insertion were performed according to the preoperative three-dimensional plan and the available posterior maxillary anatomy. In all patients included in this subgroup, the full-arch configuration consisted of two pterygoid implants placed in the posterior maxilla combined with four conventional implants placed in the anterior maxilla, for a total of six implants. After implant insertion, conical abutments were connected and healing caps were positioned before suturing, maintaining the prosthetic references required for the subsequent digital transfer.

#### 2.4.3. Dental Implant Rehabilitation (Standard, Short and Narrow)

In patients treated with dental implant-supported full-arch rehabilitation (including standard, short and narrow implants), the Impression Reference was fixed at the palatal midline before flap elevation. An intraoral scan was acquired with the Impression Reference in place to record its relationship with the mucosal anatomy and prosthetic project. A full-thickness maxillary flap was then raised to expose the residual alveolar ridge and allow implant placement under direct vision. Implant site preparation was performed according to the prosthetically driven plan. Six implants were placed in each patient and distributed along the residual maxillary arch. Standard, short, extra-short, or narrow implants were selected according to the available bone volume, ridge morphology, and anatomical limitations. The open guide was connected to the Impression Reference when required to verify prosthetic contours, implant entry points, and emergence profiles during the surgical phase. After implant placement, conical abutments were connected and healing caps were positioned. The surgical field was then sutured, preserving the references needed for the subsequent intraoperative scan and prosthetic transfer.

### 2.5. Digital Matching and Laboratory Workflow

After implant placement, the digital workflow was continued to register the final implant positions within the prosthetic project while preserving the spatial reference provided by the Impression Reference. All intraoperative digital acquisitions and dataset-matching procedures were performed by the same prosthodontist according to the standardized workflow described below. Before proceeding with the prosthetic acquisition, the relationship between the implant entry areas, expected emergence profiles, and planned prosthetic contours was verified using the customized open guide connected to the Impression Reference. Once implant placement had been completed, the healing caps were removed and scan abutments were connected to the implants. A second intraoral scan was then performed with the Impression Reference still fixed in situ. This scan recorded the position of the scan abutments, the surrounding buccal soft tissues, and the posterior anatomical reference areas of the edentulous maxilla, while maintaining the same stable reference geometry used during the previous intraoperative acquisition. After verification of the scan quality, the Impression Reference was removed and the healing caps were repositioned. A postoperative orthopantomography was obtained to assess implant positioning. The acquired intraoperative datasets were subsequently imported into dedicated digital dental software and aligned with the preoperative soft-tissue and prosthetic scans. This matching procedure allowed the final implant positions and the prosthetic reference information to be integrated into a single virtual environment. In this way, the planned intermaxillary and occlusal relationships were maintained, and the immediate screw-retained full-arch restoration could be designed according to the original prosthetic project. In the atrophic edentulous maxilla, this step was considered critical because the Impression Reference provided a stable and recognizable geometry for dataset registration throughout the surgical and prosthetic sequence.

### 2.6. Prosthetic Procedure

**Figure 10:**
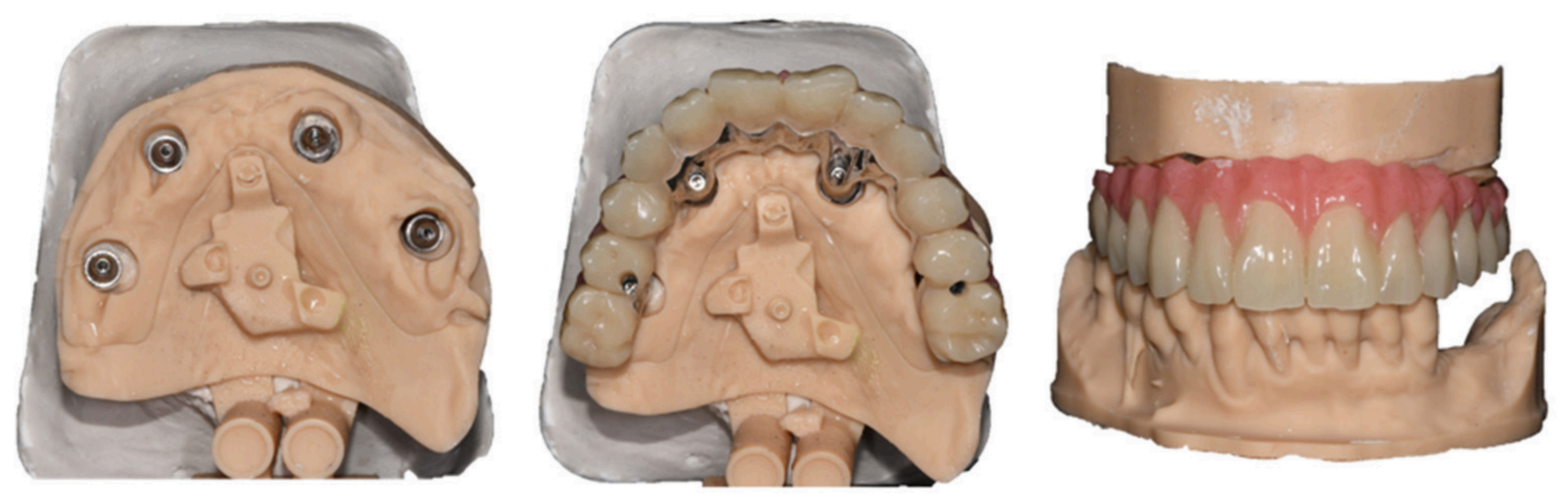
Post-surgical file matching and fabrication of the provisional prosthetic restoration.

**Figure 11:**
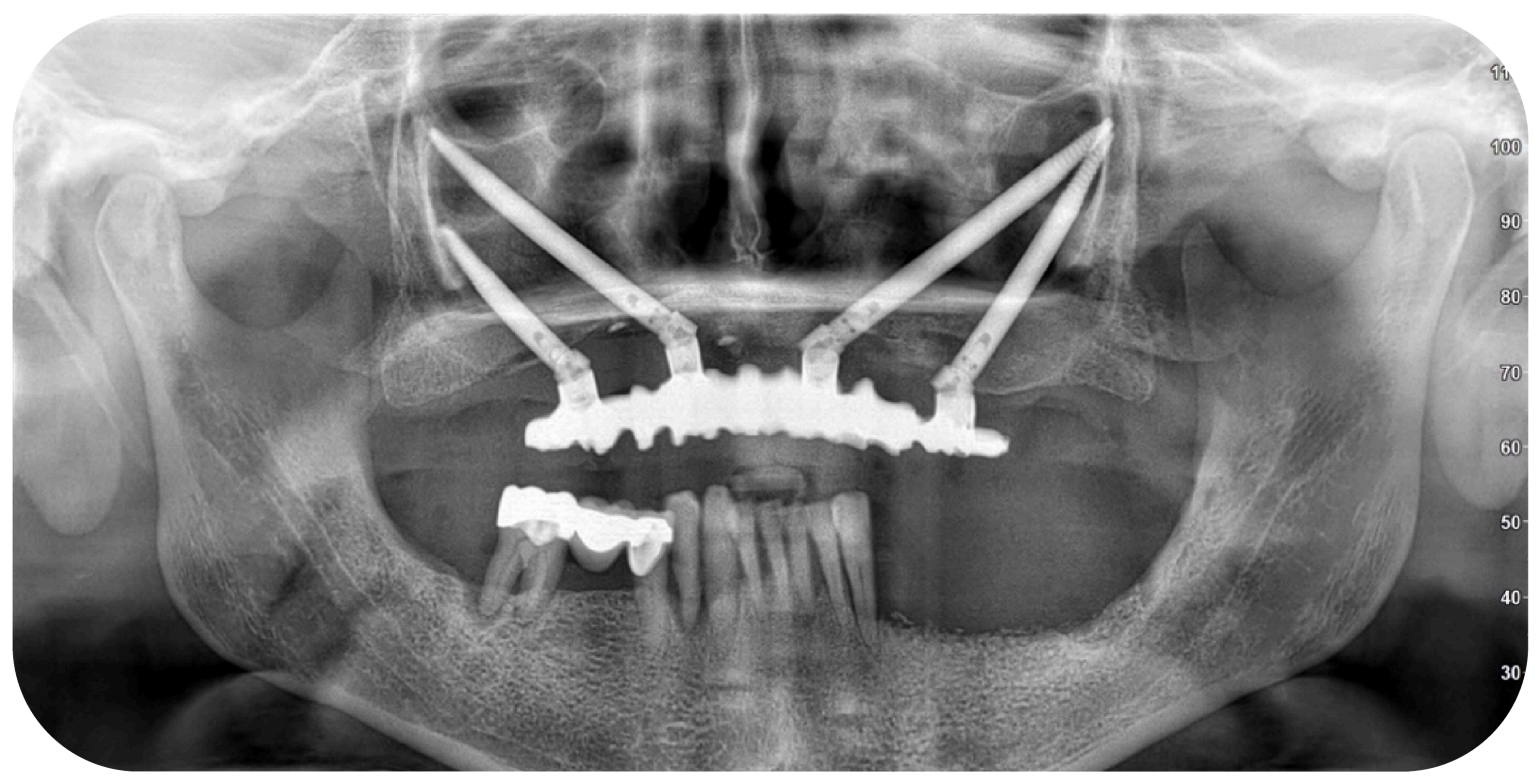
Immediate prosthetic loading.

**Figure 12:**
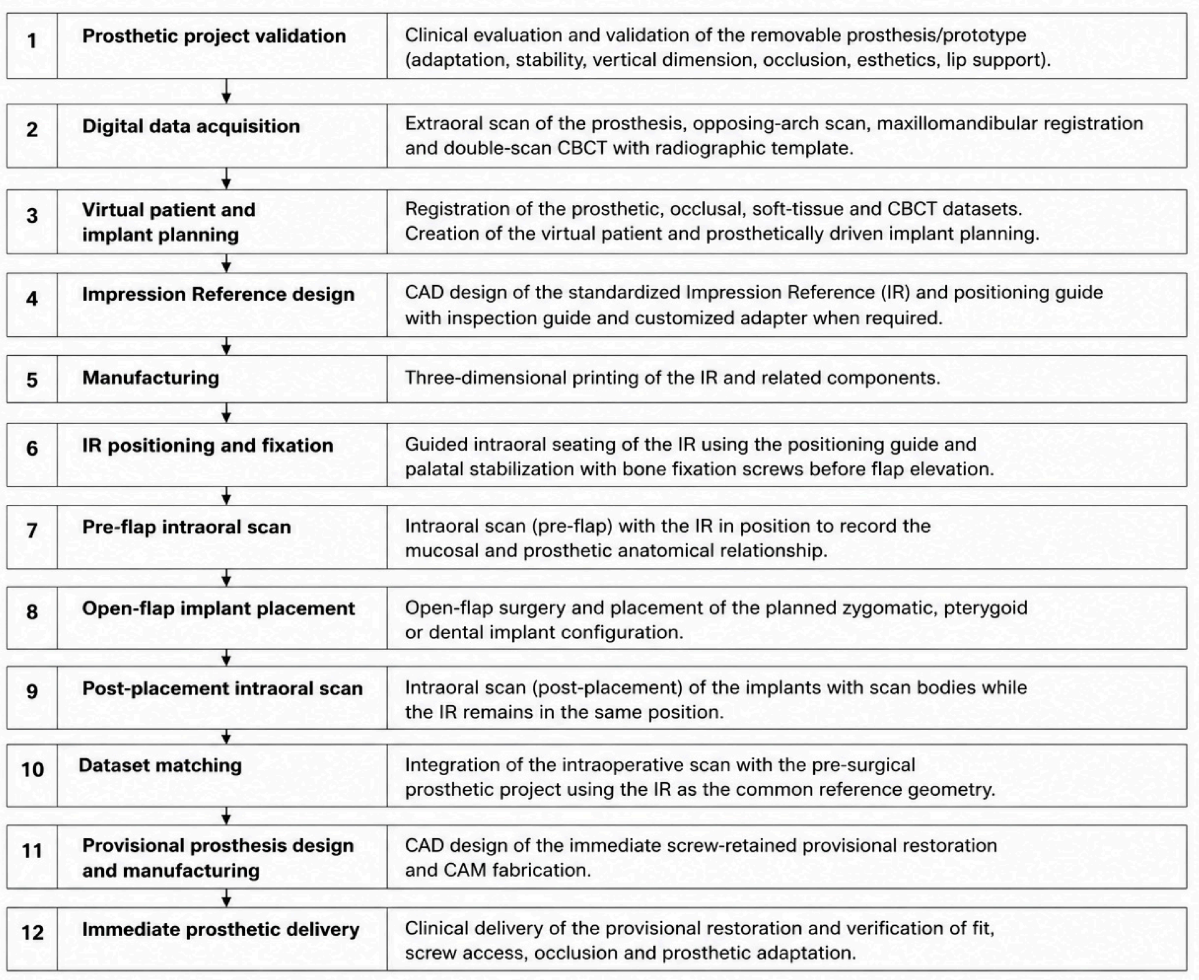
Schematic representation of the Impression Reference-assisted digital workflow.

After alignment of the intraoperative and preoperative datasets, the prosthetic workflow was continued within the digital environment. The matched files allowed for the integration of the final implant positions, peri-implant soft-tissue morphology, and prosthetic reference information into the original virtual project. On the basis of this integrated dataset, the immediate screw-retained full-arch restoration was designed according to the preoperative functional and esthetic plan. Implant emergence, prosthetic contours, occlusal relationships, and the extension of the restoration were digitally verified before finalizing the project for CAD/CAM manufacturing. When required, the prosthetic contour guide reproducing the external profile of the planned restoration was used as an additional reference to confirm the correspondence between the virtual prosthetic design and the clinical situation. The immediate prosthesis was then fabricated and delivered according to the planned loading protocol (Figure 10). At delivery, the passive fit of the restoration, screw access positions, occlusal contacts, and overall prosthetic adaptation were clinically checked. After the necessary occlusal adjustments, the restoration was screwed into place to complete the immediate full-arch rehabilitation of the edentulous maxilla (Figure 11). The complete clinical and digital sequence is summarized in Figure 12.

### 2.7. Data Collection and Outcome Measures

For each patient, demographic, clinical, procedural, and workflow-related data were retrieved from the available records. Patient names were anonymized and replaced with identification codes before data extraction. The collected variables included age, sex, rehabilitation protocol, number and distribution of implants, implant configuration, documented complications related to the index procedure, completion of the Impression Reference-assisted digital workflow, completion of dataset matching, delivery of the immediate prosthesis, clinically acceptable prosthesis fit, prosthetic congruence with the pre-surgical digital project, and compliance of the patient-specific CAD design with the nominal 0.2 mm coupling tolerance. The implant configuration was recorded as four zygomatic implants, two posterior pterygoid implants combined with four conventional anterior implants, or six dental implants distributed along the residual maxillary arch. Rehabilitation protocols were classified according to the implant-supported strategy used, including zygomatic, pterygoid, standard, short, extra-short, and narrow implants. The primary workflow-related outcomes were completion of the Impression Reference-assisted digital workflow, completion of dataset matching, and delivery of an immediate screw-retained full-arch maxillary restoration. Secondary descriptive outcomes included documented complications related to the index procedure, clinically acceptable prosthesis fit, prosthetic congruence with the pre-surgical digital project, and compliance of the CAD coupling design with the nominal 0.2 mm tolerance. Clinically acceptable prosthesis fit was assessed at prosthesis delivery using a three-level clinical protocol. First, complete seating was evaluated visually by inspecting the implant–abutment and prosthesis–abutment interfaces, as applicable, for detectable gaps, incomplete seating, rocking, or instability. Second, a Sheffield one-screw test was performed. One terminal prosthetic screw was tightened while the remaining screws were left untightened, and the contralateral and intermediate interfaces were evaluated for lifting or marginal discrepancies. The procedure was then repeated from the opposite terminal side. Radiographic verification was used to assess complete seating and identify detectable gaps at interfaces that could not be adequately inspected directly. Third, tactile sensation during sequential screw tightening was assessed, including whether the prosthesis seated progressively and uniformly without resistance, displacement, or spring-back suggestive of misfit. Clinically acceptable prosthesis fit was recorded when none of these assessments identified a detectable misfit and the restoration could be delivered without sectioning or major prosthetic correction. This outcome represented a structured clinical assessment and was not based on microscopic gap measurement, strain analysis, torque–time analysis, or another quantitative measurement of implant-level passivity. Prosthetic congruence with the pre-surgical digital project was recorded when the delivered restoration was clinically consistent with the validated prosthetic volume, tooth position, midline, lip support, emergence areas, screw-access positions, and occlusal relationships. This outcome was assessed descriptively and was not derived from a quantitative three-dimensional deviation analysis. Compliance with the nominal 0.2 mm coupling tolerance was assessed during the premanufacturing CAD verification of each patient-specific project. This variable was recorded when the designed coupling between the Impression Reference and the associated components respected the predefined tolerance. After manufacturing, component coupling was evaluated clinically by confirming complete seating and the absence of detectable instability during assembly and use. No quantitative metrological measurement of the physical discrepancy between the manufactured components was performed. The study was limited to workflow-related and immediate prosthetic-delivery outcomes. Implant survival, prosthesis survival, insertion torque, implant stability measurements, marginal bone-level changes, and long-term biological or mechanical complications were not included in the prespecified data collection because these variables and follow-up assessments were not standardized for the present workflow-oriented investigation.

### 2.8. Data Analysis

Given the descriptive nature of the present workflow feasibility case series, all data were analyzed descriptively. Demographic and clinical characteristics were summarized for the overall study population and according to the rehabilitation protocol. Age was reported as mean, median, and range. Categorical variables, including sex, rehabilitation protocol, implant configuration, documented complications related to the index procedure, completion of the Impression Reference-assisted digital workflow, completion of dataset matching, delivery of the immediate prosthesis, clinically acceptable prosthesis fit, prosthetic congruence with the pre-surgical digital project, and compliance of the CAD coupling design with the nominal 0.2 mm tolerance, were reported as counts and percentages. No inferential statistical analysis or comparisons among implant-supported rehabilitation subgroups were performed because the study was designed to evaluate the feasibility of completing the Impression Reference-assisted digital and prosthetic workflow rather than differences in implant-related or clinical outcomes.

## 3. Results

### 3.1. Study Population and Rehabilitation Protocols

This case series included twenty-six completely edentulous patients with maxillary atrophy, comprising 14 women and 12 men. The mean age was 65.2 years (median: 66.5 years), with a range of 40 to 80 years. All patients underwent maxillary full-arch rehabilitation using an open-flap, immediately loaded approach, supported by an Impression Reference-based workflow. Zygomatic implant-supported rehabilitation was performed in 16 patients, with four zygomatic implants placed in each patient. Four patients received a six-implant configuration consisting of two posterior pterygoid implants combined with four conventional implants placed in the anterior maxilla. The remaining six patients received six dental implants distributed along the residual maxillary arch: 4 × 4 mm extra-short implants in two patients, narrow-diameter implants in two patients, 5 × 5 mm short implants in one patient, and standard implants in one patient. A total of 124 implants were placed across the 26 patients. Implant-supported configurations were reported descriptively and were not compared statistically because the study was not designed to evaluate differences among rehabilitation subgroups. Demographic characteristics and implant configurations are summarized in Table 1.

### 3.2. Workflow-Related Outcomes

The Impression Reference-assisted digital workflow was completed in all 26 patients. Intraoperative digital acquisition, dataset matching, prosthetic transfer, and delivery of an immediate screw-retained full-arch maxillary restoration were achieved in every case. All patient-specific digital projects complied with the predefined nominal CAD coupling tolerance of 0.2 mm. Following manufacturing, the Impression Reference and the associated components could be completely seated and remained clinically stable during the relevant workflow steps in all cases. The nominal 0.2 mm value referred exclusively to the premanufacturing CAD design verification and did not represent a quantitative measurement of the physical discrepancy between the manufactured components or of the overall accuracy of the workflow. Successful dataset matching was recorded as completion of the planned registration procedure. No quantitative assessment of registration error, implant positional deviation, or three-dimensional transfer accuracy was performed.

### 3.3. Prosthetic Delivery and Clinical Findings

An immediate screw-retained full-arch maxillary prosthesis was delivered in all 26 patients. Clinically acceptable prosthesis fit was recorded in every case according to the predefined visual, radiographic, Sheffield one-screw test, and tactile assessment criteria. No restoration required sectioning or major prosthetic correction before delivery. Prosthetic congruence with the pre-surgical digital project was also recorded in all patients. The delivered restorations were clinically consistent with the validated prosthetic project in terms of prosthetic volume, tooth position, midline, lip support, emergence areas, screw-access positions, and occlusal relationships. No intraoperative surgical complications related to the index procedure were documented in the available clinical records. This finding should be interpreted descriptively, as complications were not assessed within a standardized prospective follow-up protocol.

## 4. Discussion

### 4.1. Main Findings

This case series describes the feasibility of an Impression Reference-assisted digital workflow for open-flap immediate loading full-arch rehabilitation of the atrophic maxilla. The workflow was completed in all included patients, with successful intraoperative digital acquisition, dataset matching, prosthetic transfer, and delivery of immediate screw-retained maxillary restorations. The contribution of the present study should be distinguished from previous technical reports on the Impression Reference concept. Earlier publications introduced the spatial-reference principle and subsequently described its application in open-flap workflows. The present case series specifically evaluated the third-generation configuration, characterized by a standardized core reference combined with patient-specific positioning, inspection, and connecting components. It also described its application across different implant-supported rehabilitation protocols rather than within a single implant-specific scenario. All patient-specific projects complied with the nominal 0.2 mm CAD coupling tolerance, and the associated components could be clinically assembled and used throughout the planned workflow. This finding reflects compliance with the predefined design requirement and should not be interpreted as a quantitative measurement of coupling accuracy, dataset-matching accuracy, or prosthetic fit. The results support the clinical feasibility of completing the Impression Reference-assisted workflow in the included patients. However, the study did not quantify registration error, implant positional deviation, prosthetic misfit, or long-term clinical outcomes.

### 4.2. Comparison with Stackable Guide Systems

Stackable guide systems have been proposed to improve the sequential transfer of the digital plan in full-arch implant rehabilitation. These systems typically rely on interconnected templates that guide multiple treatment phases, including bone reduction, implant placement, and immediate provisionalization. Yang et al. (2021) [33] described a digital workflow using a stackable surgical guide fabricated with selective laser melting, emphasizing its role in enhancing the predictability of bone reduction, implant insertion, and immediate loading of provisional prostheses. More recently, Lan et al. (2024) [34], in a scoping review, noted that stackable guides may assist the clinician from flap elevation to the delivery of a screw-retained temporary prosthesis, while also acknowledging that current evidence remains limited and further studies are needed. Although both stackable guides and the Impression Reference are intended to support continuity between the virtual plan, the surgical procedure, and prosthetic delivery, their primary functions differ. Stackable guides primarily support the sequential surgical and prosthetic execution of the treatment plan, whereas the Impression Reference is primarily intended to support dataset matching and prosthetic transfer after implant placement. This distinction is functional and should not be interpreted as evidence of superior accuracy or clinical performance.

### 4.3. Integrated Facially and Prosthetically Driven Digital Workflows

Several integrated digital workflows have been proposed to enhance the connection between esthetic analysis, prosthetic planning, implant placement, and immediate restoration in complete-arch rehabilitation. Pozzi, Arcuri, and Moy introduced the Smiling Scan technique [35], a facially driven approach that combines CBCT, optical scanning, and prosthetic data to create a virtual dental patient, thereby improving the esthetic and functional orientation of guided surgery. The same group later developed the Digital Assisted Soft Tissue Sculpturing (DASS) technique [36], which previsualizes the ideal soft- and hard-tissue interface for pink-free prostheses and prefabricates a CAD/CAM interim prosthesis to serve as a scaffold for tissue conditioning at surgery. Unlike implant-centric approaches, DASS emphasizes the interplay between bone architecture, soft-tissue contour, prosthetic emergence, and immediate restoration design. Pozzi and co-workers (2021) [37] also described workflows combining dynamic navigation with prosthetic planning and immediate loading, using real-time transfer of implant and prosthetic coordinates to facilitate delivery of digitally prefabricated complete-arch prostheses. A subsequent clinical study [38] further supported the feasibility of integrating prosthetically driven planning, navigation-guided placement, and immediate loading. These approaches share with the present Impression Reference workflow the overarching goal of reducing discrepancies between the presurgical plan, surgery, and prosthetic delivery. However, their transfer strategies differ: Smiling Scan prioritizes facial and esthetic planning; DASS focuses on soft- and hard-tissue prosthetic interface conditioning; and dynamic navigation enhances intraoperative control of implant trajectory. In contrast, the Impression Reference is designed to remain fixed during open-flap surgery, providing a stable physical landmark capturable by intraoral scanning. Its role is specifically oriented toward dataset matching and prosthetic transfer after implant placement, rather than planning integration, tissue sculpturing, or trajectory control. This distinction is particularly relevant in atrophic edentulous maxillae, where open-flap surgery alters soft-tissue morphology and undermines the reliability of preoperative mucosal references. In this context, the Impression Reference is intended to act as a physical bridge between the presurgical prosthetic project, intraoperative acquisition, and laboratory fabrication of the immediate prosthesis. In this context, the Impression Reference is intended to act as a physical link between the pre-surgical prosthetic project, intraoperative acquisition, and laboratory fabrication of the immediate restoration. These digital workflows may have complementary roles, as each is designed to address a different aspect of the relationship between virtual planning, the surgical field, and prosthetic delivery.

### 4.4. Implant Position Recording Techniques: Scan Bodies, Horizontal Scan Bodies, and Photogrammetry

A distinct group of digital techniques focuses on recording implant positions after placement. In complete-arch rehabilitations, this step is particularly critical, as intraoral scanning over long edentulous spans may be compromised by stitching errors, limited anatomical landmarks, scan-body geometry, implant distribution, and operator- or scanner-related variability. Various strategies have therefore been investigated to improve the trueness and precision of complete-arch digital implant impressions. Conventional scan-body-based protocols are widely used to transfer implant positions into the digital environment, yet their reliability depends on the design, orientation, and distribution of the scan bodies, as well as on the intraoral scanner employed. Horizontal intraoral scan bodies with occlusal geometry have been proposed to enhance accuracy in this phase. Etxaniz et al. (2025) [39] described a technique using such devices to obtain more accurate virtual casts, and Laureti et al. (2025) [40] subsequently reported that horizontal designs may improve consistency and trueness compared with conventional vertical scan bodies, although performance remains influenced by scanner and operator variables. Photogrammetry offers another strategy for complete-arch implant position recording, capturing the three-dimensional relationship among multiple implants with high accuracy while reducing some limitations of conventional intraoral scanning over edentulous spans. Cheng et al. (2024) [41], in an in vitro study, found that stereophotogrammetry showed superior trueness and precision compared with intraoral scanning, meeting misfit thresholds for implant-supported complete-arch prostheses. These techniques are highly relevant for the prosthetic phase, as they aim to optimize the digital capture of implant coordinates after placement. The Impression Reference workflow described in this case series has a different primary purpose: rather than replacing scan bodies, horizontal scan bodies, or photogrammetry, it is designed to maintain spatial continuity between the presurgical prosthetic plan, the intraoperative condition, and the laboratory workflow in open-flap immediate loading rehabilitation. Scan bodies, horizontal scan bodies, and photogrammetry primarily support implant-position recording, whereas the Impression Reference is intended to provide a spatial reference linking the pre-surgical prosthetic project, intraoperative acquisition, and prosthetic delivery. These technologies may therefore have complementary roles within the digital transfer pathway. However, unlike quantitative studies evaluating scan bodies or photogrammetry, the present case series did not measure implant-coordinate trueness, precision, or three-dimensional transfer error. The comparison is therefore functional and workflow-related and should not be interpreted as evidence of equivalent or superior accuracy.

### 4.5. Dynamic Navigation

Dynamic navigation has been introduced in implant dentistry to improve intraoperative transfer of the preoperative plan by providing real-time control of drill position, angulation, and depth. Systematic reviews report that it may enhance placement accuracy compared with freehand surgery while offering greater intraoperative flexibility than static guided protocols [42,43], a feature particularly valuable in complex anatomy, where implant trajectories must adapt to residual bone and prosthetic requirements. In complete-arch rehabilitation, Pozzi et al. (2021) combined dynamic navigation with prosthetic planning for immediate loading, noting that this approach facilitates delivery of digitally prefabricated prostheses [37]. In atrophic maxillae, dynamic navigation has also been applied to zygomatic implants, where real-time tracking helps control long and complex trajectories and reduce deviation from the planned path [44]. However, dynamic navigation and the Impression Reference address distinct steps of the digital workflow. The former primarily supports surgical execution of the planned implant trajectory; the latter provides a stable spatial reference for intraoperative scanning, dataset matching, and prosthetic transfer after implant placement. These approaches address different phases of the workflow and may potentially be used in a complementary manner. In open-flap immediate loading rehabilitation of the atrophic maxilla, dynamic navigation may assist implant positioning, while the Impression Reference supports the subsequent transfer of implant-related and prosthetic information into the laboratory workflow.

### 4.6. Limitations and Future Perspectives

This study has several limitations. First, its descriptive case-series design and the absence of a control group preclude direct comparisons with alternative digital workflows, such as stackable guide systems, photogrammetry, conventional or horizontal scan-body protocols, or dynamic navigation. In addition, the cohort included different implant-supported configurations, with a predominance of zygomatic rehabilitations. Although this heterogeneity reflects the clinical application of the workflow across different anatomical conditions, the limited and unbalanced subgroup sizes do not allow for comparisons among rehabilitation strategies. Second, the study was designed to evaluate the feasibility of completing the digital and prosthetic transfer workflow rather than its quantitative accuracy. The main outcomes were therefore recorded as binary or clinically assessed variables. Successful dataset matching indicates that the planned clinical and laboratory sequence could be completed, but it does not quantify the magnitude of the registration error. STL superimposition error, implant positional deviation, angular or linear transfer discrepancies, screw-access deviation, prosthetic-interface gaps, and mechanical strain were not measured. The available datasets were acquired for clinical treatment and not according to a standardized metrological research protocol; therefore, a retrospective quantitative accuracy analysis was not considered sufficiently reliable. The assessment of prosthesis fit and congruence with the pre-surgical prosthetic project was based on predefined clinical criteria, including visual inspection, radiographic verification, the Sheffield one-screw test, and tactile evaluation during screw tightening. However, these assessments should not be interpreted as quantitative measurements of implant-level passivity or prosthetic accuracy. Similarly, the nominal 0.2 mm value referred exclusively to the coupling tolerance incorporated and verified during the CAD design phase between the Impression Reference and the associated components. It did not represent a clinical or metrological measurement of the physical discrepancy between the manufactured components or of the overall accuracy of the workflow. Third, the investigation primarily focused on prosthetic transfer rather than on implant-level biomechanical, radiographic, or biological outcomes. Consequently, insertion torque, implant stability measurements, abutment-specific variables, and marginal bone-level changes were not systematically collected. Furthermore, the heterogeneous implant configurations limited the applicability and comparability of a single implant-level stability parameter across the entire cohort. The present findings should therefore not be interpreted as evidence regarding implant stability, osseointegration, peri-implant bone maintenance, or differences in clinical performance among the implant subgroups. Fourth, only completely edentulous maxillary cases treated with an open-flap immediate loading protocol were included. Accordingly, the findings may not be generalizable to mandibular rehabilitations, flapless procedures, partially edentulous patients, or workflows without immediate prosthetic delivery. Follow-up duration was also variable, and the study was not designed to evaluate implant or prosthesis survival, marginal bone changes, or long-term biological and mechanical complications. Finally, chairside time, laboratory time, number of appointments, production costs, and overall cost-effectiveness were not prospectively recorded. The Impression Reference workflow requires digital planning, fabrication of dedicated components and guides, additive manufacturing, intraoperative scanning, and close coordination between the clinical and laboratory teams. These requirements may initially increase planning time, costs, and the need for trained personnel and dedicated equipment. Conversely, the use of a stable spatial reference may facilitate dataset matching and prosthetic transfer and may reduce the need to repeat conventional impression procedures. These potential advantages should be evaluated in future comparative studies. Prospective controlled investigations should incorporate standardized acquisition protocols, predefined landmarks and reference surfaces, validated three-dimensional superimposition methods, quantitative prosthetic-fit assessments, chairside and laboratory time, economic analyses, patient-reported outcomes, and longer clinical follow-up. Such studies would help determine the accuracy, efficiency, cost-effectiveness, and specific indications of the Impression Reference workflow in comparison with other digital transfer strategies.

## 5. Conclusions

Within the limitations of this descriptive uncontrolled case series, the Impression Reference-assisted digital workflow could be completed in all included patients undergoing open-flap immediate loading full-arch rehabilitation of the atrophic maxilla. The technique was used to maintain a recognizable spatial reference between the pre-surgical prosthetic project, intraoperative digital acquisitions, and immediate prosthetic delivery. All patient-specific projects fulfilled the predefined nominal CAD coupling tolerance, and the associated components could be clinically assembled and used during the planned workflow. Immediate screw-retained full-arch restorations were delivered in all cases, with clinically acceptable prosthesis fit according to the predefined visual, radiographic, Sheffield-test, and tactile criteria. These findings support the clinical feasibility of the workflow in the included patients but do not provide quantitative evidence of dataset-matching accuracy, implant positional accuracy, prosthetic passivity, predictability, or superiority over alternative digital transfer strategies. Furthermore, the study was not designed to evaluate implant or prosthesis survival, marginal bone-level changes, or long-term biological and mechanical complications. Prospective controlled studies incorporating standardized three-dimensional accuracy analyses, quantitative prosthetic-fit measurements, economic and time-related outcomes, and longitudinal clinical follow-up are required to further evaluate the performance and indications of the Impression Reference-assisted workflow.

## Figures and Tables

**Table 1:** Demographic characteristics, rehabilitation protocols, and workflow-related outcomes of patients treated with open-flap immediate loading full-arch rehabilitation of the atrophic maxilla using the Impression Reference technique.

Patient ID	Age	Sex	Treated Jaw	Rehabilitation Protocol	N° of Implants	Immediate Loading	Surgical Complications	Completion of IR-Assisted Digital Workflow	Completion of Dataset Matching	Clinically Acceptable Passive Fit	Prosthetic Congruence with the Pre-Surgical Digital Plan	CAD Coupling Design Within the Nominal 0.2 mm Tolerance	Immediate Prosthesis Delivered	Available Clinical Record Period
P01	66	F	Maxilla	Zygomatic Implants	4	✓	–	✓	✓	✓	✓	✓	✓	5 years
P02	64	F	Maxilla	Zygomatic Implants	4	✓	–	✓	✓	✓	✓	✓	✓	1 year
P03	72	M	Maxilla	Zygomatic Implants	4	✓	–	✓	✓	✓	✓	✓	✓	2 years
P04	76	M	Maxilla	Zygomatic Implants	4	✓	–	✓	✓	✓	✓	✓	✓	3 years
P05	80	F	Maxilla	Zygomatic Implants	4	✓	–	✓	✓	✓	✓	✓	✓	4 years
P06	56	F	Maxilla	5 × 5 Short Implants	6	✓	–	✓	✓	✓	✓	✓	✓	1 year
P07	78	M	Maxilla	4 × 4 Extra-short Implants	6	✓	–	✓	✓	✓	✓	✓	✓	3 years
P08	66	M	Maxilla	Pterygoid Implants	2 pterygoid + 4 dental	✓	–	✓	✓	✓	✓	✓	✓	2 years
P09	61	M	Maxilla	Zygomatic Implants	4	✓	–	✓	✓	✓	✓	✓	✓	3 years
P10	67	F	Maxilla	Zygomatic Implants	4	✓	–	✓	✓	✓	✓	✓	✓	2 years
P11	74	M	Maxilla	Narrow Implants	6	✓	–	✓	✓	✓	✓	✓	✓	1 year
P12	68	F	Maxilla	Zygomatic Implants	4	✓	–	✓	✓	✓	✓	✓	✓	2 years
P13	70	F	Maxilla	Zygomatic Implants	4	✓	**–**	✓	✓	✓	✓	✓	✓	1 year
P14	67	M	Maxilla	Pterygoid Implants	2 pterygoid + 4 dental	✓	**–**	✓	✓	✓	✓	✓	✓	2 years
P15	63	F	Maxilla	Zygomatic Implants	4	✓	**–**	✓	✓	✓	✓	✓	✓	3 years
P16	75	F	Maxilla	Zygomatic Implants	4	✓	**–**	✓	✓	✓	✓	✓	✓	5 years
P17	76	M	Maxilla	Zygomatic Implants	4	✓	**–**	✓	✓	✓	✓	✓	✓	1 year
P18	40	F	Maxilla	4 × 4 Extra-short Implants	6	✓	**–**	✓	✓	✓	✓	✓	✓	1 year
P19	65	F	Maxilla	Pterygoid Implants	2 pterygoid + 4 dental	✓	**–**	✓	✓	✓	✓	✓	✓	3 years
P20	40	M	Maxilla	Zygomatic Implants	4	✓	**–**	✓	✓	✓	✓	✓	✓	2 years
P21	59	M	Maxilla	Zygomatic Implants	4	✓	**–**	✓	✓	✓	✓	✓	✓	5 years
P22	62	F	Maxilla	Zygomatic Implants	4	✓	**–**	✓	✓	✓	✓	✓	✓	4 years
P23	62	F	Maxilla	Pterygoid Implants	2 pterygoid + 4 dental	✓	**–**	✓	✓	✓	✓	✓	✓	2 years
P24	76	M	Maxilla	Standard Implants	6	✓	**–**	✓	✓	✓	✓	✓	✓	3 years
P25	44	M	Maxilla	Narrow Implants	6	✓	**–**	✓	✓	✓	✓	✓	✓	1 year
P26	68	F	Maxilla	Zygomatic Implants	4	✓	**–**	✓	✓	✓	✓	✓	✓	5 years

## Data Availability

All relevant data are available from the corresponding author upon reasonable request.

## Abbreviations

The following abbreviations are used in this manuscript:

CAD/CAM	Computer-aided design/manufacturing
CBCT	Cone-Beam Computed Tomography
DASS	Digital Assisted soft tissue sculpturing
DIBINEM	Department of Biomedical and Neuromotor Sciences
DLP	Digital Light Processing
IR	Impression Reference
SLM	Selective Laser Melting
3D	Three-Dimensional

## References

[1] Schwarz F., Schär A., Nelson K., Fretwurst T., Flügge T., Ramanauskaite A., Trimpou G., Sailer I., Karasan D., Fehmer V. (2021). Recommendations for Implant-Supported Full-Arch Rehabilitations in Edentulous Patients: The Oral Reconstruction Foundation Consensus Report. Int. J. Prosthodont..

[2] Morton D., Gallucci G., Lin W., Pjetursson B., Polido W., Roehling S., Sailer I., Aghaloo T., Albera H., Bohner L. (2018). Group 2 ITI Consensus Report: Prosthodontics and implant dentistry. Clin. Oral Implants Res..

[3] Spencer K.R. (2018). Implant based rehabilitation options for the atrophic edentulous jaw. Aust. Dent. J..

[4] Ali S.A., Karthigeyan S., Deivanai M., Kumar A. (2014). Implant rehabilitation for atrophic maxilla: A review. J. Indian Prosthodont. Soc..

[5] Michelinakis G., Apostolakis D., Kamposiora P., Papavasiliou G., Özcan M. (2021). The direct digital workflow in fixed implant prosthodontics: A narrative review. BMC Oral Health.

[6] Nulty A. (2024). A literature review on prosthetically designed guided implant placement and the factors influencing dental implant success. Br. Dent. J..

[7] Froimovici F.-O., Butnărașu C.C., Montanari M., Săndulescu M. (2024). Fixed Full-Arch Implant-Supported Restorations: Techniques Review and Proposal for Improvement. Dent. J..

[8] Abduo J., Rasaie V. (2025). Digital Workflows in Prosthodontics. Aust. Dent. J..

[9] Fokas G., Vaughn V.M., Scarfe W.C., Bornstein M.M. (2018). Accuracy of linear measurements on CBCT images related to presurgical implant treatment planning: A systematic review. Clin. Oral Implants Res..

[10] Li J., Joda T., Revilla-León M., Saleh M.H.A., Chen Z., Wang H.-L. (2024). Recommendations for successful virtual patient-assisted esthetic implant rehabilitation: A guide for optimal function and clinical efficiency. J. Esthet. Restor. Dent..

[11] Wang J., Wang B., Liu Y.Y., Luo Y.L., Wu Y.Y., Xiang L., Yang X.M., Qu Y.L., Tian T.R., Man Y. (2024). Recent Advances in Digital Technology in Implant Dentistry. J. Dent. Res..

[12] Li J., Chen Z., Dong B., Wang H.L., Joda T., Yu H. (2020). Registering Maxillomandibular Relation to Create a Virtual Patient Integrated with a Virtual Articulator for Complex Implant Rehabilitation: A Clinical Report. J. Prosthodont..

[13] Wismeijer D., Joda T., Flügge T., Fokas G., Tahmaseb A., Bechelli D., Bohner L., Bornstein M., Burgoyne A., Caram S. (2018). Group 5 ITI Consensus Report: Digital technologies. Clin. Oral Implants Res..

[14] Azevedo L., Marques T., Karasan D., Fehmer V., Sailer I., Correia A., Gómez-Polo M. (2024). Effect of splinting scan bodies on the trueness of complete arch digital implant scans with 5 different intraoral scanners. J. Prosthet. Dent..

[15] Carneiro Pereira A.L., Souza Curinga M.R., Melo Segundo H.V., da Fonte Porto Carreiro A. (2023). Factors that influence the accuracy of intraoral scanning of total edentulous arches rehabilitated with multiple implants: A systematic review. J. Prosthet. Dent..

[16] Al-Nawas B., Aghaloo T., Aparicio C., Bedrossian E., Brecht L., Brennand-Roper M., Chow J., Davó R., Fan S., Jung R. (2023). ITI consensus report on zygomatic implants: Indications, evaluation of surgical techniques and long-term treatment outcomes. Int. J. Implant Dent..

[17] Pellegrino G., Tarsitano A., Ratti S., Ceccariglia F., Gessaroli M., Barausse C., Tayeb S., Felice P. (2025). Zygomatic implants for rehabilitation of patients with oncologic and congenital defects: A case series. J. Cranio-Maxillofac. Surg..

[18] Testori T., Clauser T., Rapani A., Artzi Z., Avila-Ortiz G., Barootchi S., Bressan E., Chiapasco M., Cordaro L., Decker A. (2024). Indications for implant-supported rehabilitation of the posterior atrophic maxilla: A multidisciplinary consensus among experts in the field utilising the modified Delphi method. Int. J. Oral Implantol..

[19] Felice P., Bonifazi L., Pistilli R., Trevisiol L., Pellegrino G., Nocini P.F., Barausse C., Tayeb S., Bersani M., D’Agostino A. (2024). Zygomatic implants in the rehabilitation of severe maxillary atrophy: A retrospective study of 274 zygomatic implants with a mean follow-up period of 7.5 years. Int. J. Oral Implantol..

[20] Pala K., Neugebauer J., Buser D., Brunello G., Chmielewski K., Chowdhary R., Cordaro L., Hosokawa R., Jensen O., Jung U.W. (2026). Consensus Report of Group 2 of the 1st Global Consensus for Clinical Guidelines for the Rehabilitation of the Edentulous Maxilla: Zygomatic, Standard-Length, and Short Implant-Supported Prostheses. Clin. Oral Implants Res..

[21] Aalam A.A., Krivitsky-Aalam A., Kurtzman G.M., Mahesh L. (2023). The severely atrophic maxilla: Decision making with zygomatic and pterygoid dental implants. J. Oral Biol. Craniofacial Res..

[22] Strauss F.J., Brunello G., Thoma D.S., Kopp I., Stilwell C., Jung R.E., Wang H.L., Schwarz F. (2026). 1st Global Consensus for Clinical Guidelines for the Rehabilitation of the Edentulous Maxilla: A Single-Round Survey on Standard, Short, and Zygomatic Implant-Supported Prostheses. Clin. Oral Implants Res..

[23] Araujo R.Z., Santiago Júnior J.F., Cardoso C.L., Condezo A.F.B., Moreira Júnior R., Curi M.M. (2019). Clinical outcomes of pterygoid implants: Systematic review and meta-analysis. J. Cranio-Maxillofac. Surg..

[24] D’Amario M., Orsijena A., Franco R., Chiacchia M., Jahjah A., Capogreco M. (2024). Clinical achievements of implantology in the pterygoid region: A systematic review and meta-analysis of the literature. J. Stomatol. Oral Maxillofac. Surg..

[25] Guida L., Annunziata M., Esposito U., Sirignano M., Torrisi P., Cecchinato D. (2020). 6-mm-short and 11-mm-long implants compared in the full-arch rehabilitation of the edentulous mandible: A 3-year multicenter randomized controlled trial. Clin. Oral Implants Res..

[26] Rosa A., Pujia A.M., Arcuri C. (2023). Complete Full Arch Supported by Short Implant (<8 mm) in Edentulous Jaw: A Systematic Review. Appl. Sci..

[27] Kostunov J., Giannakopoulos N.N., Rammelsberg P., Kappel S. (2025). Two- Versus Four-Narrow-Implant-Retained Dentures With Immediate-Loaded Anterior Implants: 9 Years Randomized Clinical Trial. Clin. Implant Dent. Relat. Res..

[28] Agliardi E., Clericò M., Ciancio P., Massironi D. (2010). Immediate loading of full-arch fixed prostheses supported by axial and tilted implants for the treatment of edentulous atrophic mandibles. Quintessence Int..

[29] Lanis A., Alvarez Del Canto O., Barriga P., Polido W.D., Morton D. (2019). Computer-guided implant surgery and full-arch immediate loading with prefabricated-metal framework-provisional prosthesis created from a 3D printed model. J. Esthet. Restor. Dent..

[30] Chan M.H., Holmes C. (2015). Contemporary “All-on-4” concept. Dent. Clin. N. Am..

[31] Mazzoni A., Pellegrino G., Breccia C., Di Bene P., Mattoli R., Bonifazi L., Barausse C., Felice P. (2023). A new impression reference technique to simplify the digital workflow for immediate loading zygomatic implant-supported rehabilitation. Int. J. Oral Implantol..

[32] Pellegrino G., Anselmo G., Barausse C., Ratti S., Breccia C., Mancuso E., Giudice A., Di Bene P., Felice P. (2024). Impression Reference Technique for the Open Flap Digital Workflow in the Immediate Loading Rehabilitation of the Upper and Lower Jaws. Prosthesis.

[33] Yang J.W., Liu Q., Yue Z.G., Hou J.X., Afrashtehfar K.I. (2021). Digital Workflow for Full-Arch Immediate Implant Placement Using a Stackable Surgical Guide Fabricated Using SLM Technology. J. Prosthodont..

[34] Lan R., Marteau C., Mense C., Silvestri F. (2024). Current knowledge about stackable guides: A scoping review. Int. J. Implant Dent..

[35] Pozzi A., Arcuri L., Moy P.K. (2018). The smiling scan technique: Facially driven guided surgery and prosthetics. J. Prosthodont. Res..

[36] Pozzi A., Arcuri L., Block M.S., Moy P.K. (2021). Digital assisted soft tissue sculpturing (DASS) technique for immediate loading pink free complete arch implant prosthesis. J. Prosthodont. Res..

[37] Pozzi A., Hansson L., Carosi P., Arcuri L. (2021). Dynamic navigation guided surgery and prosthetics for immediate loading of complete-arch restoration. J. Esthet. Restor. Dent..

[38] Pozzi A., Arcuri L., Carosi P., Nardi A., Kan J. (2021). Clinical and radiological outcomes of novel digital workflow and dynamic navigation for single-implant immediate loading in aesthetic zone: 1-year prospective case series. Clin. Oral Implants Res..

[39] Etxaniz O., Amezua X., Jauregi M., Solaberrieta E. (2025). Improving the accuracy of complete arch implant intraoral digital scans by using horizontal scan bodies with occlusal geometry: A dental technique. J. Prosthet. Dent..

[40] Laureti A., Marques T., Pitta J., Fehmer V., Sailer I., Pozzi A., Azevedo L. (2025). Influence of Horizontal Intraoral Scan Bodies on the Trueness of Digital Impressions for Complete-Arch Prostheses on Four Implants: An In Vitro Evaluation. Clin. Oral Implants Res..

[41] Cheng J., Zhang H., Liu H., Li J., Wang H.L., Tao X. (2024). Accuracy of edentulous full-arch implant impression: An in vitro comparison between conventional impression, intraoral scan with and without splinting, and photogrammetry. Clin. Oral Implants Res..

[42] Pellegrino G., Ferri A., Del Fabbro M., Prati C., Gandolfi M.G., Marchetti C. (2021). Dynamic Navigation in Implant Dentistry: A Systematic Review and Meta-analysis. Int. J. Oral Maxillofac. Implants.

[43] Wang F., Bornstein M.M., Hung K., Fan S., Chen X., Huang W., Wu Y. (2018). Application of Real-Time Surgical Navigation for Zygomatic Implant Insertion in Patients With Severely Atrophic Maxilla. J. Oral Maxillofac. Surg..

[44] Pellegrino G., Lizio G., Basile F., Stefanelli L.V., Marchetti C., Felice P. (2020). Dynamic Navigation for Zygomatic Implants: A Case Report about a Protocol with Intraoral Anchored Reference Tool and an Up-To-Date Review of the Available Protocols. Methods Protoc..

